# A nationwide cohort study on diabetes severity and risk of Parkinson disease

**DOI:** 10.1038/s41531-023-00462-8

**Published:** 2023-01-27

**Authors:** Kyungdo Han, Bongsung Kim, Seung Hwan Lee, Mee Kyoung Kim

**Affiliations:** 1 Department of Statistics and Actuarial Science, Soongsil University, Seoul, 06978 Korea; 2 Division of Endocrinology and Metabolism, Department of Internal Medicine, Seoul St. Mary’s Hospital, College of Medicine, The Catholic University of Korea, Seoul, 06591 Korea; 3 Division of Endocrinology and Metabolism, Department of Internal Medicine, Yeouido St. Mary’s Hospital, College of Medicine, The Catholic University of Korea, Seoul, 07345 Korea

**Keywords:** Risk factors, Epidemiology

## Abstract

There is growing evidence that patients with type 2 diabetes mellitus (DM) have an increased risk of developing Parkinson’s disease (PD) and share similar dysregulated pathways. We aimed to determine whether the risk of PD increases as diabetes progresses among patients with type 2 DM. Using a nationally representative database from the Korean National Health Insurance System, 2,362,072 individuals (≥40 years of age) with type 2 DM who underwent regular health checkups during 2009–2012 were followed up until the end of 2018. The diabetes severity score parameters included the number of oral hypoglycemic agents, diabetes duration, insulin use, or presence of chronic kidney disease, diabetic retinopathy, or cardiovascular disease. Each of these characteristics was scored as one unit of diabetes severity and their sum was defined as a diabetes severity score from 0–6. We identified 17,046 incident PD cases during the follow-up. Each component of the diabetes severity score showed a similar intensity for the risk of PD. Compared with subjects with no parameters, HR values (95% confidence intervals) of PD were 1.09 (1.04–1.15) in subjects with one diabetes severity score parameter, 1.28 (1.22–1.35) in subjects with two parameters, 1.55 (1.46–1.65) in subjects with three parameters, 1.96 (1.82–2.11) in subjects with four parameters, 2.08 (1.83–2.36) in subjects with five parameters, and 2.78 (2.05–3.79) in subjects with six parameters. Diabetes severity was associated with an increased risk of developing PD. Severe diabetes may be a risk factor for the development of PD.

## Introduction

There is growing evidence that patients with type 2 diabetes mellitus (DM) have an increased risk of developing Parkinson’s disease (PD) and share similar dysregulated pathways, which suggests the presence of common underlying pathological mechanisms^1–3^. Type 2 DM develops from insulin resistance, leading to a variety of detrimental effects on metabolism and inflammation. Similar dysregulation of glucose and energy metabolism are early events in the pathogenesis of sporadic PD. Insulin receptors are found in the basal ganglia and substantia nigra and insulin plays an essential role in regulating neuronal survival and growth, dopaminergic transmission, and maintenance of synapses^1^. Several genetic and environmental risk factors for the incidence of PD have been noted in recent studies, which have also suggested that metabolic syndrome, chronic kidney diseases (CKD), or cardiovascular diseases (CVD) are important risk factors for PD^2–7^. Because CKD or CVD could be a diabetes-related complication, we hypothesized that the risk of PD increases as diabetes progresses.

Assessing diabetes severity is important and could help identify people in need of targeted therapies and healthcare services^8–10^. Here, the severity of diabetes refers to diabetes duration, treatment status, and diabetes complications. The severity of diabetes may be evaluated by blood glucose status according to laboratory values, such as fasting blood glucose (FBG) and glycosylated hemoglobin A1c (HbA1c). However, it is difficult to assess diabetes severity using single values because these values can change and tend to wax and wane over time. As a general treatment flow in non-insulin-dependent states, such as type 2 DM, if exercise or diet does not improve glycemic control, mono- or dual therapy is started^8,9^. Moreover, if treatment control is not possible with monotherapy, patients must be treated with two or more drugs. Therefore, severity is also evaluated in terms of the number of oral hypoglycemic agents (OHAs)^8,9^. Insulin treatment is mostly prescribed for patients with type 2 DM who become insulin-deficient and/or have failed other OHAs, which is referred to as advanced diabetes^10^. Patients with type 2 DM who need insulin therapy are considered to display one of the indicators of diabetes severity. The current study investigated the association of diabetes severity with PD development using large-scale cohort data from the Korean general population.

## Results

### Baseline characteristics

During a median follow-up of 7.2 years (interquartile range, 6.0–8.1 years), there were 17,046 (0.72%) cases of incident PD. The baseline characteristics of subjects with incident PD were older age, more males, nonsmokers, higher prevalence of CKD, DR, or CVD, and more likely to receive insulin treatment or more oral antidiabetes medications (Table 1). Patients with type 2 DM who developed PD had lower levels of FBG and eGFR. The use of metformin, sulfonylurea, thiazolidinediones, or α-glucosidase inhibitors was higher in patients with PD. There was no difference in the use of dipeptidyl-peptidase 4 (DPP4) inhibitors depending on the presence of PD.

**Table 1:** Study subjects’ baseline characteristics.

	Parkinson’s disease		*P* value
	No	Yes	
*N*	2,345,026	17,046	
Age (years)	59.06 ± 10.69	68.2 ± 7.59	<0.001
Sex (male)	1,373,727 (58.6)	8099 (47.5)	<0.0001
Body mass index (kg/m^2^)	25.02 ± 3.31	24.79 ± 3.13	<0.001
Smoking			<0.001
Non-smoker	1,335,654 (56.96)	12,344 (72.42)	
Ex-smoker	444,289 (18.95)	2818 (16.53)	
Current smoker	565,083 (24.1)	1884 (11.05)	
Alcohol drinking	226,713 (9.67)	774 (4.54)	<0.001
Regular exercise	495,348 (21.12)	3469 (20.35)	0.014
Income (lower 25%)	496,804 (21.19)	3275 (19.21)	<0.001
Systolic BP (mmHg)	129.25 ± 15.9	130 ± 16.2	<0.001
Diastolic BP (mmHg)	79.05 ± 10.27	77.77 ± 10.28	<0.001
Fasting glucose (mg/dL)	144.04 ± 46.51	137.53 ± 46.26	<0.001
eGFR (ml/min/1.73 m^2^)	84.3 ± 35.59	76.69 ± 28.22	<0.001
Baseline TC (mg/dL)	196.17 ± 42.51	190.58 ± 42.58	<0.001
Dyslipidemia	1,011,422 (43.1)	8195 (48.1)	<0.001
Hypertension	1,383,458 (59.0)	12066 (70.8)	<0.001
Chronic kidney disease	280,481 (12.0)	3788 (22.2)	<0.001
Diabetic retinopathy	200,063 (8.5)	2740 (16.1)	<.0001
Myocardial infarction	53,746 (2.3)	619 (3.6)	<0.001
Stroke	173,656 (7.4)	3010 (17.7)	<0.001
Depression	243,096 (10.4)	3803 (22.3)	<0.001
Duration of diabetes ≥5 years	763,547 (32.6)	8171 (47.9)	<0.001
Pharmacologic therapy for DM			
Insulin	194,658 (8.3)	2402 (14.1)	<0.001
Number of OHAs ≥3	331,346 (14.1)	3392 (19.9)	<0.001
Medication			
Metformin	1,026,078 (43.8)	9394 (55.1)	<0.001
Sulfonylurea	968,067 (41.3)	9421 (55.3)	<0.001
DPPIV-inhibitors	154,635 (6.6)	1115 (6.5)	0.781
TZD	152,638 (6.5)	1370 (8.0)	<0.001
AGI	276,524 (11.8)	3109 (18.2)	<0.001

Values are expressed as mean ± standard deviation, or number (%).

*BP* blood pressure, *eGFR* estimated glomerular filtration rate, *TC* total cholesterol, *OHAs* oral hypoglycemic agents, *DPPIV-inhibitors* dipeptidyl-peptidase IV inhibitors, *TZD* thiazolidinedione, *AGI* alpha-glucosidase inhibitors.

### Incidence and risk of PD according to diabetes severity score parameters

Each of the diabetes severity score parameters showed an association with a similar intensity to the risk of PD, even after adjustment for confounding factors (Table 2). Subjects with insulin use had an HR of 1.36 (95% CI: 1.30–1.42) for PD compared with those treated without insulin. The use of three or more OHAs and a diabetes duration of ≥5 years were also significantly associated with an increased risk of PD (Model 2, HR, 1.15; 95% CI: 1.11–1.20 and HR, 1.23, 95% CI: 1.19–1.28, respectively). The risk of PD increased significantly in subjects with CKD compared with subjects without CKD (adjusted HR, 1.20; 95% CI: 1.15–1.25). Patients with DR were associated with a significantly higher risk of PD than diabetics without DR (adjusted HR, 1.35; 95% CI: 1.30–1.41), even after adjusting for age, sex, BMI, alcohol intake, smoking, regular exercise, hypertension, dyslipidemia, and depression. There was also a significantly increased risk of PD in subjects with CVD (adjusted HR, 1.38; 95% CI: 1.33–1.45). The results of the sex-specific analysis are presented in Supplementary Tables 1, 2.

**Table 2:** The risk of Parkinson’s disease stratified according to the diabetes severity parameters.

		Events (*n*)	Incidence rate (per 1000 person-years)	Model 1	Model 2
Duration of diabetes	<5 years	8875	0.813	1 (ref.)	1 (ref.)
	≥5 years	8171	1.543	1.37 (1.33, 1.41)	1.23 (1.19, 1.28)
Number of oral hypoglycemic agents	<3	13,654	0.983	1 (ref.)	1 (ref.)
	≥3	3392	1.460	1.31 (1.26, 1.32)	1.15 (1.11, 1.20)
Use of insulin	No	14,644	0.982	1 (ref.)	1 (ref.)
	Yes	2402	1.853	1.62 (1.55, 1.69)	1.36 (1.30, 1.42)
Chronic kidney disease	No	13,258	0.925	1 (ref.)	1 (ref.)
	Yes	3788	2.016	1.21 (1.17, 1.26)	1.20 (1.15, 1.25)
Diabetic retinopathy	No	14,306	0.965	1 (ref.)	1 (ref.)
	Yes	2740	1.984	1.56 (1.50, 1.63)	1.35 (1.30, 1.41)
Cardiovascular disease	No	13,615	0.921	1 (ref.)	1 (ref.)
	Yes	3431	2.392	1.60 (1.54, 1.66)	1.38 (1.33, 1.45)

Model 1: adjusted for age and sex.

Model 2: adjusted for age, sex, BMI, alcohol drinking, smoking, regular exercise, hypertension, dyslipidemia, and depression.

### Incidence and risk of PD according to the diabetes severity score

**Fig. 1:**
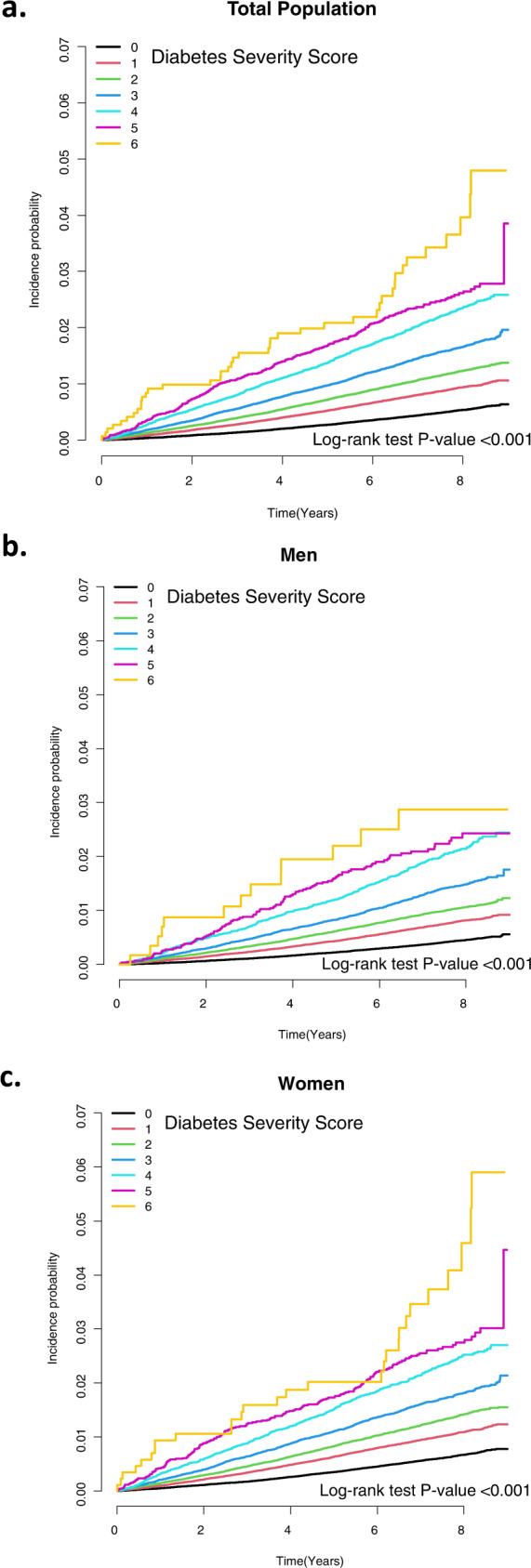
Kaplan–Meier estimates for the cumulative incidence of Parkinson’s disease according to the diabetes severity score (0–6). The probability of incident Parkinson’s disease according to the diabetes severity score was analyzed for the total population (**a**), men (**b**), and women (**c**).

The Kaplan–Meier curve (Fig. 1) shows the incidence probability of PD according to the diabetes severity score compared with the group without any diabetes severity score (score, 0). PD incidence was positively correlated with the score of diabetes severity (log-rank test, *P* < 0.001). The HR for patients with incident PD compared to patients without any diabetes severity score parameters gradually increased with the number of parameters (Table 3). After adjusting for possible confounding factors, the HR values (95% CI) of PD were 1.09 (1.04–1.15) in subjects with one diabetes severity score parameter, 1.28 (1.22–1.35) in subjects with two parameters, 1.55 (1.46–1.65) in subjects with three parameters, 1.96 (1.82–2.11) in subjects with four parameters, 2.08 (1.83–2.36) in subjects with five parameters, and 2.78 (2.05–3.79) in subjects with six parameters compared with subjects with no parameters. Subjects with three diabetes severity score parameters were at 55% higher risk of PD and those with five diabetes severity score parameters were at 108% higher risk, compared to those without any parameters.

**Table 3:** The risk of Parkinson’s disease according to the diabetes severity score.

	Total	Events (*n*)	Incidence rate (per 1000 person-years)	Model 1	Model 2
Total population					
0	1,221,120	5378	0.640	1 (ref.)	1 (ref.)
1	583,678	4667	1.153	1.22 (1.17, 1.27)	1.09 (1.04, 1.15)
2	332,552	3485	1.527	1.48 (1.41, 1.54)	1.28 (1.22, 1.35)
3	153,646	2141	2.076	1.84 (1.75, 1.93)	1.55 (1.46, 1.65)
4	55,942	1052	2.927	2.43 (2.28, 2.60)	1.96 (1.82, 2.11)
5	13,663	282	3.385	2.69 (2.39, 3.04)	2.08 (1.83, 2.36)
6	1471	41	4.846	3.67 (2.70, 4.99)	2.78 (2.05, 3.79)
Men					
0	769,185	2833	0.538	1 (ref.)	1 (ref.)
1	328,400	2236	0.990	1.22 (1.15, 1.29)	1.09 (1.01, 1.17)
2	176,952	1581	1.320	1.44 (1.35, 1.53)	1.24 (1.15, 1.35)
3	75,135	905	1.837	1.77 (1.64, 1.91)	1.48 (1.36, 1.63)
4	25,592	426	2.682	2.35 (2.12, 2.61)	1.89 (1.69, 2.12)
5	5964	105	3.047	2.57 (2.11, 3.12)	1.98 (1.61, 2.42)
6	598	13	3.986	3.24 (1.88, 5.58)	2.44 (1.41, 4.22)
Women					
0	451,935	2545	0.81148	1 (ref.)	1 (ref.)
1	255,278	2431	1.35926	1.22 (1.15, 1.29)	1.10 (1.02, 1.19)
2	155,600	1904	1.75594	1.50 (1.41, 1.59)	1.32 (1.23, 1.42)
3	78,511	1236	2.29444	1.88 (1.75, 2.01)	1.62 (1.49, 1.75)
4	30,350	626	3.12195	2.47 (2.26, 2.70)	2.02 (1.83, 2.23)
5	7699	177	3.62248	2.76 (2.37, 3.22)	2.17 (1.85, 2.54)
6	873	28	5.3852	3.93 (2.71, 5.71)	3.02 (2.08, 4.40)

Model 1: adjusted for age and sex.

Model 2: adjusted for age, sex, BMI, alcohol drinking, smoking, regular exercise, hypertension, dyslipidemia, and depression.

### Effect of fasting blood glucose levels in type 2 diabetes

The FBG levels associated with the lowest risk of PD were 120–130 mg/dL (Supplementary Table 3). There was a J-shaped association between the risk of PD and glycemic control, with both the too-low and high FBG showing a higher risk of PD. Multivariable-adjusted HRs (95% CI) of PD associated with FBG 140–149, 150–159, 160–169, 170–179, 180–189, 190–199, and ≥ 200 mg/dL were 1.10 (1.01–1.19), 1.10 (1.01–1.20), 1.10 (0.99–1.34), 1.21 (1.08–1.34), 1.20 (1.07–1.36), 1.17 (1.02–1.34), and 1.23 (1.14–1.34), respectively, compared with the FBG 120–129 mg/dL. The risk of PD was also increased when FBG was <100 mg/dL (HR, 1.09; 95% CI: 1.02–1.16). We found that FBG <100 mg/dL and ≥140 mg/dL were associated with an increased risk of PD. So, we assigned 0 points to FBG 100–139 mg/dl and 1 point to FBG <100 mg/dl or ≥ 140 mg/dl. We then summed these to give a diabetes severity score ranging from 0 to 7 points. When assessing the severity score ranging from 0–7, including the category of FBG levels, multivariable-adjusted HRs for PD risk increased continuously and linearly with increasing severity scores (Supplementary Table 4).

### Association of PD risk and diabetes severity in subgroup analysis

**Fig. 2:**
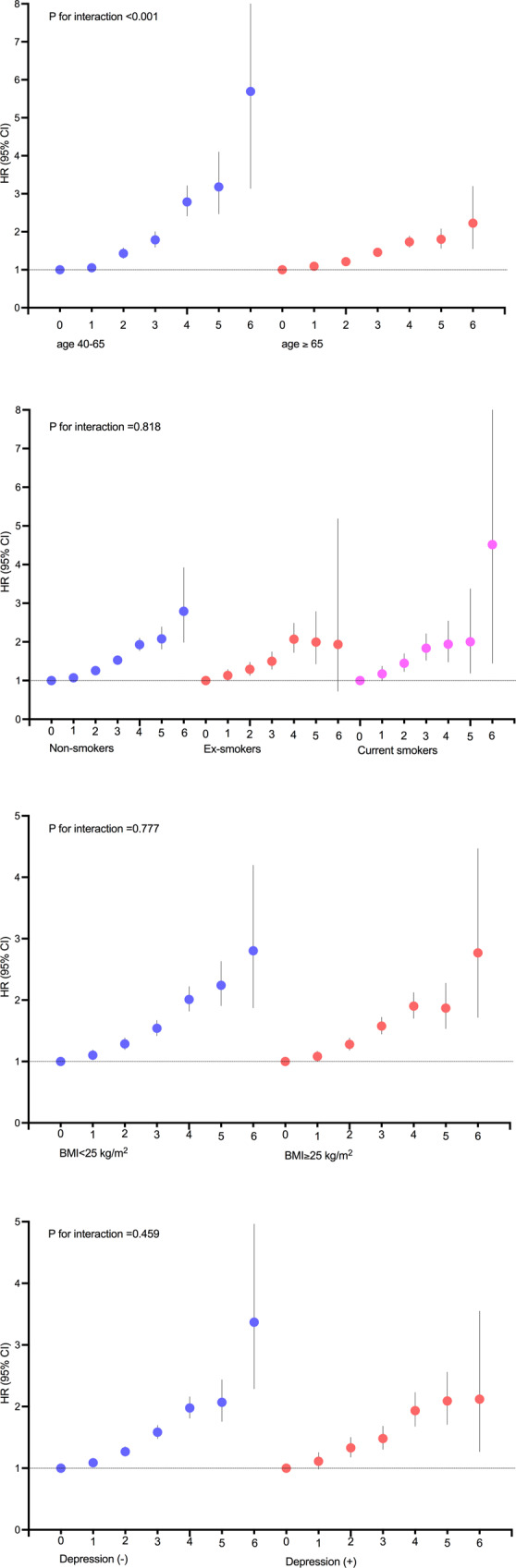
Subgroup analyses of the association between the diabetes severity score and risk of Parkinson’s disease stratified by age, smoking status, obesity, and the presence of depression. Hazard ratios and 95% confidence intervals of Parkinson’s disease by the diabetes severity score. Adjusted for age, sex, body mass index, alcohol intake, smoking, regular exercise, hypertension, dyslipidemia, and depression.

We conducted a stratified analysis using age, smoking status, BMI category, and the presence of depression. A significant association between the diabetes severity score and the risk of PD was observed in all subgroups. There was a significant interaction between age and diabetes severity on the risk of PD (*P* for interaction <0.001). Compared with subjects >65 years of age, we observed a greater increase in the HR of PD with increasing diabetic severity scores in subjects aged 40–65 years old. The risk of PD according to the diabetes severity score was similar regardless of smoking, obesity, or depression (Fig. 2; *P* for interaction >0.05).

## Discussion

Diabetes severity, as measured by diabetic complication status, treatment complexity, and duration of diabetes, was strongly associated with an increased risk for PD. All characteristics indicative of diabetes severity had an additive association with the risk of PD in patients with type 2 DM. The coexistence of conditions such as retinopathy, nephropathy, CVD, the complexity of diabetes treatments, or the long duration of diabetes was more strongly associated with PD risk than the presence of a single condition alone. People with a diabetes severity score of 4 or higher had a more than doubled risk of PD compared with those with a score of 0, and those with a score of 6 had a 2.78-fold increased risk of PD. These associations became stronger for younger diabetic patients. Our population-based, large-scale cohort study suggests that advanced diabetes may be a risk factor for the development of PD.

The incidence of PD is proportional to the degree of exposure to hyperglycemia and the duration of diabetes^2^. Compared with the nondiabetes group, the adjusted HR was 1.038 in the impaired fasting glucose group, 1.185 in the diabetes duration <5 years group, and 1.618 in the diabetes duration ≥5 years group^2^. Other studies have also identified a long diabetes duration as a crucial factor that significantly increases the risk of PD in patients with DM^11^. Recent epidemiological studies have individually evaluated the association of the risk of PD with various diabetes medications. A recent UK study demonstrated that the use of DPP4 inhibitors and/or glucagon-like peptide-1 (GLP-1) mimetics is associated with a lower rate of PD compared to the use of other oral antidiabetic drugs^12^. GLP-1 receptor stimulation in the central nervous system (CNS) may impact insulin receptor signaling pathways and may also enhance neuronal survival pathways. GLP-1 receptor agonizts were introduced into Korea in 2011; therefore, there were few GLP-1 receptor agonizts users during this study period. The addition of thiazolidinediones, DPP4 inhibitors, or GLP-1 mimetics suggested as drugs to prevent PD may achieve more effective glycemic control, thus limiting the damaging effects of excess glycation on overall brain function. The status of patients requiring multiple antidiabetic medications should be interpreted as reflecting their insulin resistance and high glycemic burden. The Danish study found that any antidiabetic drug or insulin therapy was associated with an increased risk of PD^13^.

There is growing evidence that a process analogous to peripheral insulin resistance occurs in the brains of PD patients, which suggests that loss of insulin signaling may contribute to the development of the pathological features of PD^1^. Although no data yet exists from human studies testing exogenous insulin in patients with PD, clinical trials in Alzheimer’s disease (AD) have shown the potential utility of using insulin to restore insulin signaling defects^1^. Trials of intranasal insulin administered to patients with mild cognitive dysfunction and early AD led to improvements in verbal memory and cognition^14^. In our study, insulin use was associated with an increased risk of PD. Progression to treatment intensification is a sign of disease progression; therefore, progression to insulin therapy may represent the severity of disease progression in type 2 DM.

We found that each of the DM complications, including retinopathy, nephropathy, or CVD was associated with an increased risk of PD. Patients with diabetes without DR were reported to have a 1.54-fold increased risk of PD compared with patients without diabetes, whereas patients with DR had a 2.39-fold increased risk of PD compared with patients without diabetes^3^. It has been reported that patients with DR were associated with a significantly higher risk of PD than non-diabetic patients or in diabetes without DR. The retina is part of the CNS and uses dopamine as a key neurotransmitter^3^. Loss of dopaminergic amacrine cells in the retina was also found in diabetic mice. A 3-year follow-up study based on the Longitudinal Health Insurance Database in Taiwan reported that uremia in patients is associated with a 1.81-fold higher risk of developing parkinsonism than in patients without uremia^15^. The coexistence of chronic renal dysfunction and proteinuria is positively associated with the risk of PD^6^. Proteinuria has been recognized as a primary marker for renal damage because it can be detected earlier than the apparent decline in eGFR. Scholars examining the association of CKD and PD have suggested potential pathways explaining a future increased risk for PD, where the renin–angiotensin system (RAS), oxidative stress, and inflammation have a significant role. CKD conditions involve an altered RAS due to increased renin, which leads to increased circulating angiotensin II levels^16^. In conditions of an altered blood–brain barrier, angiotensin II reaches brain regions, binds to angiotensin receptors, and induces oxidative stress and microglia activation, followed by neuronal damage. It has been reported that a previous stroke is associated with an approximately twofold higher risk for PD in the Chinese population (HR, 1.94; 95% CI: 1.39–2.69)^17^. Cerebral ischemia potentially activates the dopaminergic pathway due to decreased expression of nicotinic acetylcholine receptors and plays a role in the clinical expression and deterioration of idiopathic PD symptoms. Small vessel disease is more common in patients with diabetes and may contribute to the appearance of “vascular parkinsonism,” which may have been mistakenly diagnosed as PD^12,18^. The South Korean government has operated a rare intractable diseases (RID) registration program (V-code), which includes 167 conditions including PD. The V124 code yields a major benefit to PD patients who only must pay 10% of all medical expenditures; therefore, assigning this code to a patient requires careful and complicated documentation. To assign the V124 code to patients, diagnostic MRI findings and major parkinsonism syndromes, including tremor, bradykinesia, rigidity, and postural instability should be examined and confirmed by a neurologist. In Korea, this enables an accurate diagnosis of PD to be made^19^.

Notably, FBG <100 mg/dL and ≥140 mg/dL were associated with an increased risk of PD. The association was significant even after adjustment for confounders. Hypoglycemia, along with hyperglycemia, is also known to be an indicator of the severity of diabetes^10^. There was a J-shaped association between the risk of PD and glycemic control, with both the too-low and high FBG showing a higher risk of PD. Both high and low HbA1c levels outside the window of euglycemia have been associated with faster motor symptom progression in PD^20^. The magnitude of risk in our study was greater in younger subjects whereby genetic factors may exert relatively more of an effect. The association in elderly patients may be the consequence of disrupted insulin signaling secondary to lifestyle and environmental factors causing cumulative pathogenic brain changes^21^. Whether attributable to genetic predisposition, environmental factors, or both, disrupted brain insulin signaling could lead to shared dysregulated cellular pathways, including neuroinflammation, microglia activation, mitochondrial dysfunction, and increased oxidative stress, which ultimately promote synuclein aggregation and contribute to the development of PD^21^. This concept is supported by a higher risk among those with diabetes complications and those with long duration of type 2 DM. Our study is characterized by a focus only on patients with type 2 DM. Within patients with type 2 DM, we found the risk of PD increased in severe or complicated type 2 DM.

There is still no universal agreement on the optimal data needed to create a reliable diabetes severity measure, despite the presence of some diabetes-specific severity indices^10^. The clinical manifestations of more severe diabetes are a consequence of diverse and complex pathophysiological processes affecting different organ systems over time, making it difficult for a single severity measure to capture this complexity adequately^10^. Prior studies on diabetes severity used a grading system based on diabetes-related complications and glycemic control measures^10^. Some studies considered the type or patterns of prescribed therapies as a proxy for higher diabetes severity^8,22,23^. The complexity of treatment and the use of multiple antidiabetic drugs are also used as indicators of severity^8,22,23^. It was also reported that the use of more than three OHAs was associated with a 13% increased risk of new DM complications compared to the monotherapy (Relative risk 1.13, 95% CI 1.10–1.17)^24^. A short duration of diabetes was usually defined as 0–6 years and a long duration of diabetes was generally defined as ≥8–10 years. There is always some uncertainty about the duration of a diabetic state prior to diagnosis, and with a known duration over 5–6 years, reversal of type 2 DM after bariatric surgery or intensive weight management intervention was less likely to occur^22,25^. It was reported that for each 5-year increase in the duration of diabetes, the multiple adjusted risks of macrovascular events and all-cause death were increased by 13 and 15%, respectively^26^. Moreover, we previously reported a 62% increased risk of PD in the diabetic duration ≥5 years group compared to the non-diabetic group^2^. Patients with type 2 DM requiring insulin therapy are considered to display one of the indicators of severe diabetes^10,27^. In one study, the severity of type 2 DM was categorized into four levels using an automated algorithm based on two domains: insulin use and the presence of DM complications^27^. Therefore, the severity of DM was evaluated in terms of the number of OHAs ≥3, duration of diabetes ≥5 years, or insulin use.

This study had some limitations. First, this was an observational study. Therefore, the association found between the stratification parameters and endpoints may not be causal. To minimize the possible effects of reverse causality, we excluded those individuals with incident PD during the first year of follow-up. Second, because the identification of PD was based on nationwide claims data, we could not obtain clinical information and imaging findings. Third, the influence of other diabetic complications (e.g., diabetic neuropathy) could not be evaluated properly. The insurance claim database does not provide accurate diagnostic information for diabetic neuropathy. We did not study data on HbA1c or postprandial glucose levels, since it is difficult to conduct these tests for all participants in a mass screening program. Fourth, patients with severe or complicated diabetes are more likely to have increased contact with healthcare and this could result in bias from increased medical surveillance. Lastly, diabetes severity score was calculated by assigning equal weighting to all components in the overall score. This could be a limitation in this study without providing the reliability and validity of the scoring scheme. Both the Diabetes Complications Severity Index (DCIS), capturing the type and severity of complications, and a simple count of diabetes complications were similarly strongly associated with mortality and hospitalization in patients with type 2 DM^28^. According to the study on diabetes severity in the UK, the predictive value of simple count scores using 29 diabetes-related domains was slightly higher than the severity-weighted score^29^.

In conclusion, our study consolidates the evidence that diabetes severity constitutes risk factors for PD in a population with DM. The strength of this study is the accurate diagnosis of PD using the V124 special code along with the ICD-10 diagnostic code based on the characteristics of the Korean health insurance system. Moreover, we conducted this study with a large number of subjects using a well-established and validated longitudinal national database with a 7-year follow-up. However, longer follow-up duration may reduce the possibility of reverse causation and support a temporal relationship between diabetes severity and the risk of PD. A novel finding is that the risk of PD increases as the diabetes severity score, which is measured by the increased complexity of diabetes medications, the duration of diabetes, and the presence of complications. Careful monitoring of neurological symptoms related to PD seems to be helpful for patients with progressive diabetes. Further studies are warranted to examine whether control of diabetes and its complications can decrease the risk of PD.

## Methods

### Data source and study population

The National Health Insurance Service (NHIS) computerized database includes data for most of the Korean population, such as all insurance claims and most medical information. The NHIS database contains information regarding healthcare use, demographic characteristics, diagnostic data, procedures, and drug prescription records. The database also contains data from the registration program for rare and intractable diseases (V-code). The NHIS covers employees and regional insurance subscribers. All examinees were requested to have biannual health checkups, except nonoffice workers who are employee subscribers (annual health checkups). The results from these health examinations are compiled into preventive health checkup datasets, which constitute the largest nationwide cohort database of laboratory information in Korea^30–33^.

From the NHIS database, we selected individuals with type 2 DM aged 40 years and older who had undergone health examinations provided by the NHIS at least once from January 1, 2009, to December 31, 2012. We excluded subjects with any missing data and those who were diagnosed with PD prior to enrollment. To avoid confounding by preexisting disease and to minimize the possible effects of reverse causality, those with a new diagnosis of PD during the first year of follow-up or those who died during the first year of follow-up were also excluded. A total of 2,362,072 subjects (1,381,826 men and 980,246 women) were included in the study population and were followed until their date of death or December 31, 2018, whichever occurred first (Supplementary Fig. 1). This study was approved by the Institutional Review Board of the Soongsil University (IRB approval number: SSU-202003-HR-201). Deidentified information was used for analysis; therefore, informed consent was not required.

### Primary outcome

The primary outcome was the first recording of a diagnosis of PD after the index date, as identified by the International Classification of Disease (10th Revision [ICD-10]) code (G20) and the registration code (V124) for PD, which are assigned by neurologists or neurosurgeons. Since 2006, the South Korean government has operated a rare intractable diseases (RID) registration program (V-code). Patients who met the diagnostic criteria with physician certification were offered up to a 90% copayment reduction after RID program registration. Because the NHIS could refuse to pay hospital costs if a diagnosis did not meet specific criteria, cases are reviewed by medical institutions prior to submission to the NHIS and a reliable diagnosis can be presumed^30^.

### Data collection and definitions

The health examination provided by NHIS includes anthropometric and laboratory measurements. The general medical examination includes a survey of medical history, family history, lifestyle factors, blood pressure measurement, blood sampling, and urinalysis. Blood samples for the measurement of serum glucose and lipid levels were obtained after an overnight fast. Body mass index (BMI) was calculated as weight in kilograms divided by the square of height in meters. Information on smoking and alcohol consumption (heavy alcohol consumption defined as ≥30 g/day) was obtained using a questionnaire. Type 2 DM was defined according to the ICD-10 codes E11–14 for type 2 DM as either the principal diagnosis or the first to fourth additional diagnoses, and the prescription of one antidiabetic drug in each year or fasting glucose level ≥126 mg/dL. CKD was defined as an estimated glomerular filtration rate (eGFR) <60 mL/min/1.73 m^2^. CVD was defined as prior myocardial infarction or prior stroke, diagnosed with one or more inpatient or outpatient records of ICD-10 codes within 3 years before the index date. Diabetic retinopathy (DR) was defined by the ICD-10 code H36.0 during hospitalization or this code having been recorded at least twice in an outpatient setting among patients with type 2 DM. DR was defined as an ICD-10 code within 3 years before the index date.

### Definition of diabetes severity score

The diabetes severity score parameters used in this study were: the complexity of diabetes medications (i.e., insulin use or multiple OHAs), longer duration of diabetes, DR, diabetes-related renal complications (i.e., CKD), or presence of CVD. Each of these characteristics was treated as one unit of diabetes severity and their sum was defined as the diabetes severity score (0–6). If the number of OHAs in use was ≥3, a diabetes severity score of 1 was recorded, and if the diabetes duration was ≥5 years, a diabetes severity score of 1 was recorded. For example, a subject who uses insulin and has CKD has a diabetes severity score of 2. To identify the effect of blood sugar itself on incident PD, we also analyzed the risk of PD according to the 10 mg/dL interval of FBG levels.

### Statistical analysis

Statistical analyses were performed using SAS software (version 9.4; SAS Institute, Cary, NC, USA), and a *P* value <0.05 was considered significant. The baseline characteristics of the subjects are presented as the mean ± standard deviation or *n* (%). Subjects were classified into seven groups according to their diabetes severity score. The incidence rate of PD was calculated by dividing the number of incident cases by 1000 person-years. The cumulative incidence of primary outcomes according to the diabetes severity score was presented using unadjusted Kaplan–Meier curves, and the log-rank test was performed to analyze differences between groups. Cox proportional hazards analyses were performed to evaluate the association of diabetes severity score or its stratification parameters with incident PD, and hazard ratios (HRs) and 95% confidence intervals (CIs) were calculated. Model 1 was adjusted for age and sex. Model 2 was further adjusted for smoking status, alcohol intake, regular exercise, BMI, hypertension, dyslipidemia, and depression. Given the exploratory nature of this study, no adjustments were made for multiple comparisons. The potential effect modification by age, sex, obesity, and depression was evaluated using stratified analysis and interaction testing using a likelihood ratio test.

### Reporting summary

Further information on research design is available in the Nature Research Reporting Summary linked to this article.

## Supplementary information

Supplementary tables, figure

Reporting Summary

## Data Availability

The datasets for this study are owned by the Korea NHIS (https://nhiss.nhis.or.kr/). There are no current sharing agreements, and data were held under a data use contract with the Korea NHIS.

## Code availability

No previously unreported custom computer code or algorithm was used to generate results in this study.
