# Supplementary material for: A nationwide cohort study on diabetes severity and risk of Parkinson disease
Source: NPJ Parkinsons Dis. 2023 Jan 27;9:11. doi: 10.1038/s41531-023-00462-8 (PMC9883517; doi:10.1038/s41531-023-00462-8)

Supplementary Table 1. The risk of Parkinson disease stratified according to the diabetes severity parameters in Men

		Events (n)	Incidence rate (per 1000 person-years)	Model 1	Model 2
Duration of diabetes	< 5 years	4378	0.665	1 (ref.)	1 (ref.)
	≥ 5 years	3721	1.322	1.35 (1.29, 1.41)	1.21 (1.15, 1.28)
Number of Oral hypoglycemic agents	< 3	6553	0.802	1 (ref.)	1 (ref.)
	≥ 3	1546	1.252	1.31 (1.24, 1.38)	1.15 (1.08, 1.22)
Use of insulin	No	7067	0.808	1 (ref.)	1 (ref.)
	Yes	1032	1.567	1.55 (1.45, 1.65)	1.30 (1.21, 1.39)
Chronic kidney disease	No	6728	0.786	1 (ref.)	1 (ref.)
	Yes	1371	1.625	1.13 (1.06, 1.20)	1.09 (1.02, 1.17)
Diabetic retinopathy	No	6944	0.793	1 (ref.)	1 (ref.)
	Yes	1155	1.784	1.54 (1.45, 1.64)	1.33 (1.25, 1.43)
Cardiovascular disease	No	6504	0.752	1 (ref.)	1 (ref.)
	Yes	1595	2.106	1.59 (1.50, 1.68)	1.38 (1.29, 1.47)

Model 1: adjusted for age and sex

Model 2: adjusted for age, sex, BMI, alcohol drinking, smoking, regular exercise, hypertension, dyslipidemia and depression

Supplementary Table 2. The risk of Parkinson disease stratified according to the diabetes severity parameters in Women

		Events (n)	Incidence rate (per 1000 person-years)	Model 1	Model 2
Duration of diabetes	< 5 years	4497	1.040	1 (ref.)	1 (ref.)
	≥ 5 years	4450	1.795	1.38 (1.32, 1.44)	1.25 (1.19, 1.31)
Number of Oral hypoglycemic agents	< 3	7101	1.243	1 (ref.)	1 (ref.)
	≥ 3	1846	1.697	1.30 (1.23, 1.37)	1.15 (1.09, 1.22)
Use of insulin	No	7577	1.229	1 (ref.)	1 (ref.)
	Yes	1370	2.150	1.65 (1.55, 1.74)	1.40 (1.32, 1.48)
Chronic kidney disease	No	6530	1.132	1 (ref.)	1 (ref.)
	Yes	2417	2.335	1.30 (1.24, 1.37)	1.29 (1.22, 1.36)
Diabetic retinopathy	No	7362	1.213	1 (ref.)	1 (ref.)
	Yes	1585	2.159	1.55 (1.47, 1.63)	1.36 (1.29, 1.44)
Cardiovascular disease	No	7111	1.161	1 (ref.)	1 (ref.)
	Yes	1836	2.710	1.61 (1.53, 1.69)	1.41 (1.33, 1.49)

Model 1: adjusted for age and sex

Model 2: adjusted for age, sex, BMI, alcohol drinking, smoking, regular exercise, hypertension, dyslipidemia and depression

Supplementary Table 3. The risk of Parkinson disease by category of fasting blood glucose (FBG) among patients with type 2 diabetes mellitus

FBG	Events (n)	Incidence rate (per 1000 person-years)	Model 1	Model 2
< 100 mg/dl	2566	1.59	1.22 (1.15, 1.29)	1.09 (1.02, 1.16)
100 – 110	1581	1.44	1.16 (1.09, 1.24)	1.03 (0.96, 1.10)
110 – 120	1804	1.40	1.18 (1.11, 1.25)	1.05 (0.97, 1.12)
120 – 129	2445	0.96	1 (ref.)	1 (ref.)
130 – 139	2604	0.87	0.99 (0.93, 1.04)	1.04 (0.96, 1.12)
140 – 149	1635	0.92	1.03 (0.96, 1.09)	1.10 (1.01, 1.19)
150 – 159	1069	0.92	1.04 (0.97, 1.12)	1.10 (1.01, 1.20)
160 – 169	722	0.91	1.04 (0.96, 1.13)	1.10 (0.99, 1.21)
170 – 179	563	0.98	1.14 (1.04, 1.25)	1.21 (1.08, 1.34)
180 – 189	414	0.94	1.14 (1.02, 1.26)	1.20 (1.07, 1.36)
190 – 199	314	0.92	1.15 (1.02, 1.29)	1.17 (1.02, 1.34)
≥ 200 mg/dl	1329	0.85	1.18 (1.10, 1.26)	1.23 (1.14, 1.34)

Model 1: adjusted for age and sex
Model 2: adjusted for age, sex, BMI, alcohol drinking, smoking, regular exercise, hypertension, dyslipidemia and depression

Supplementary Table 4. The risk of Parkinson disease according to the diabetes severity score (0-7) including fasting blood glucose category

		Events (n)	Incidence rate (per 1000 person-years)	Model 1	Model 2
Fasting blood glucose	100-139 mg/dl	8434	1.062	1 (ref.)	1 (ref.)
	<100mg/dl or ≥ 140 mg/dl	8612	1.043	1.06 (1.03, 1.09)	1.10(1.06, 1.14)
Diabetes Severity Score					
	0	3068	0.686	1 (ref.)	1 (ref.)
	1	4774	0.811	1.07 (1.02, 1.12)	1.06 (0.99, 1.14)
	2	3746	1.231	1.24 (1.19, 1.31)	1.16 (1.08, 1.234
	3	2813	1.638	1.55 (1.48, 1.64)	1.40 (1.31, 1.49)
	4	1646	2.158	1.90 (1.78, 2.01)	1.64 (1.52, 1.76)
	5	779	2.994	2.47 (2.28, 2.68)	2.05 (1.87, 2.24)
	6	188	3.233	2.53 (2.18, 2.93)	2.00 (1.72, 2.34)
	7	32	5.551	4.13 (2.92, 5.85)	3.19 (2.25, 4.54)

Model 1: adjusted for age and sex

Model 2: adjusted for age, sex, BMI, alcohol drinking, smoking, regular exercise, hypertension, dyslipidemia and depression

Supplementary Figure 1. Flow chart of the study population

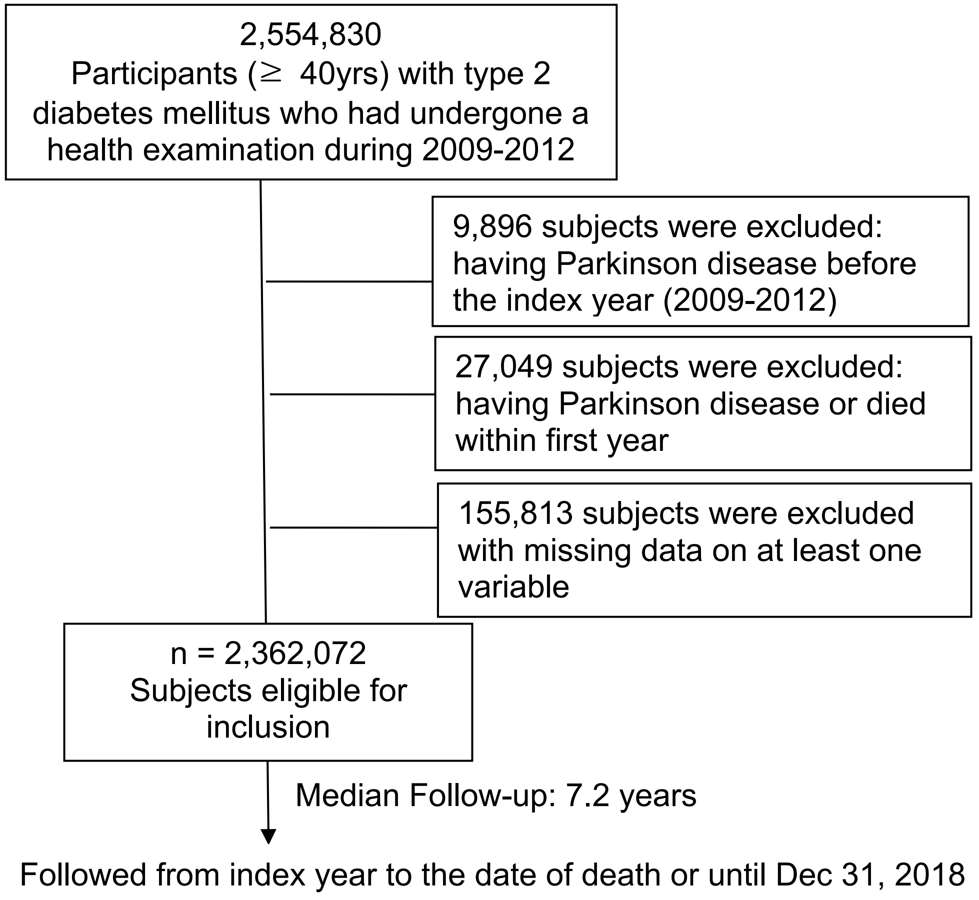

Supplement: Supplementary file 1 — Supplementary tables, figure [file 41531_2023_462_MOESM1_ESM.pdf]
