# Supplementary material for: A nationwide cohort study on diabetes severity and risk of Parkinson disease
Source: NPJ Parkinsons Dis. 2023 Jan 27;9:11. doi: 10.1038/s41531-023-00462-8 (PMC9883517; doi:10.1038/s41531-023-00462-8)
Supplement: Supplementary file 2 — Reporting Summary [file 41531_2023_462_MOESM2_ESM.pdf]

nature portfolio

Corresponding author(s): Mee Kyoung Kim

Last updated by author(s): Jan 17, 2023

Reporting Summary

Nature Portfolio wishes to improve the reproducibility of the work that we publish. This form provides structure for consistency and transparency in reporting. For further information on Nature Portfolio policies, see our Editorial Policies and the Editorial Policy Checklist.

Statistics

For all statistical analyses, confirm that the following items are present in the figure legend, table legend, main text, or Methods section.

n/a	Confirmed	
☐	☒	The exact sample size (n) for each experimental group/condition, given as a discrete number and unit of measurement
☐	☒	A statement on whether measurements were taken from distinct samples or whether the same sample was measured repeatedly
☐	☒	The statistical test(s) used AND whether they are one- or two-sided<br>*Only common tests should be described solely by name; describe more complex techniques in the Methods section.*
☐	☒	A description of all covariates tested
☐	☒	A description of any assumptions or corrections, such as tests of normality and adjustment for multiple comparisons
☐	☒	A full description of the statistical parameters including central tendency (e.g. means) or other basic estimates (e.g. regression coefficient) AND variation (e.g. standard deviation) or associated estimates of uncertainty (e.g. confidence intervals)
☐	☒	For null hypothesis testing, the test statistic (e.g. F, t, r) with confidence intervals, effect sizes, degrees of freedom and P value noted<br>*Give P values as exact values whenever suitable.*
☒	☐	For Bayesian analysis, information on the choice of priors and Markov chain Monte Carlo settings
☒	☐	For hierarchical and complex designs, identification of the appropriate level for tests and full reporting of outcomes
☒	☐	Estimates of effect sizes (e.g. Cohen's d, Pearson's r), indicating how they were calculated

Our web collection on statistics for biologists contains articles on many of the points above.

Software and code

Policy information about availability of computer code

Data collection	No software was used.
Data analysis	No software was used.

For manuscripts utilizing custom algorithms or software that are central to the research but not yet described in published literature, software must be made available to editors and reviewers. We strongly encourage code deposition in a community repository (e.g. GitHub). See the Nature Portfolio guidelines for submitting code & software for further information.

Data

Policy information about availability of data

All manuscripts must include a data availability statement. This statement should provide the following information, where applicable:

- Accession codes, unique identifiers, or web links for publicly available datasets
- A description of any restrictions on data availability
- For clinical datasets or third party data, please ensure that the statement adheres to our policy

The datasets for this study are owned by the Korea NHIS (https://nhiss.nhis.or.kr/). There are no current sharing agreements, and data are held under a data use contract with the Korea NHIS.

Human research participants

Policy information about studies involving human research participants and Sex and Gender in Research.

Reporting on sex and gender	We reported sex-based analyses (Supplemental Table 1, supplemental Table 2).
Population characteristics	See above
Recruitment	The National Health Insurance Service (NHIS) computerized database includes data for most of the Korean population. From the NHIS database, we selected individuals with type 2 DM aged 40 years and older who had undergone health examinations provided by the NHIS at least once from January 1, 2009, to December 31, 2012.
Ethics oversight	the Institutional Review Board of the Soongsil University (IRB approval number: SSU-202003-HR-201)

Note that full information on the approval of the study protocol must also be provided in the manuscript.

Field-specific reporting

Please select the one below that is the best fit for your research. If you are not sure, read the appropriate sections before making your selection.

☐ Life sciences ☒ Behavioural & social sciences ☐ Ecological, evolutionary & environmental sciences

For a reference copy of the document with all sections, see nature.com/documents/nr-reporting-summary-flat.pdf

Behavioural & social sciences study design

All studies must disclose on these points even when the disclosure is negative.

Study description	Retrospective cohort study
Research sample	The National Health Insurance Service (NHIS) computerized database includes data for most of the Korean population, such as all insurance claims and most medical information. The NHIS database contains information regarding health-care use, demographic characteristics, diagnostic data, procedures, and drug prescription records. The database also contains data from the registration program for rare and intractable diseases (V-code). The NHIS covers employees and regional insurance subscribers. All examinees were requested to have biannual health checkups, except non-office workers who are employee subscribers (annual health checkups). The results from these health examinations are compiled into preventive health checkup datasets, which constitutes the largest nationwide cohort database of laboratory information in Korea.
Sampling strategy	Our study was conducted for the purpose of exploratory data analysis using an established retrospective cohort rather than confirmatory analysis. No sample-size calculation was performed.
Data collection	Deidentified information was used for analysis.
Timing	From the NHIS database, we selected individuals with type 2 DM aged 40 years and older who had undergone health examinations provided by the NHIS at least once from January 1, 2009, to December 31, 2012.
Data exclusions	We excluded subjects with any missing data and those who were diagnosed with PD prior to enrollment (Supplemental Figure 1).
Non-participation	No participants dropped out/declined participation.
Randomization	Participants were not allocated into experimental groups.

Reporting for specific materials, systems and methods

We require information from authors about some types of materials, experimental systems and methods used in many studies. Here, indicate whether each material, system or method listed is relevant to your study. If you are not sure if a list item applies to your research, read the appropriate section before selecting a response.

Materials & experimental systems

n/a	Involved in the study
☒	☐ Antibodies
☒	☐ Eukaryotic cell lines
☒	☐ Palaeontology and archaeology
☒	☐ Animals and other organisms
☒	☐ Clinical data
☒	☐ Dual use research of concern

Methods

n/a	Involved in the study
☒	☐ ChIP-seq
☒	☐ Flow cytometry
☒	☐ MRI-based neuroimaging
